# Supplementary material for: Defects in Cell Wall Differentiation of the Arabidopsis Mutant rol1-2 Is Dependent on Cyclin-Dependent Kinase CDK8
Source: Cells. 2021 Mar 19;10(3):685. doi: 10.3390/cells10030685 (PMC8003768; doi:10.3390/cells10030685)
Supplement: Supplementary file 1 [file cells-10-00685-s001.pdf]

Supplementary Data

Suppl Figure S1

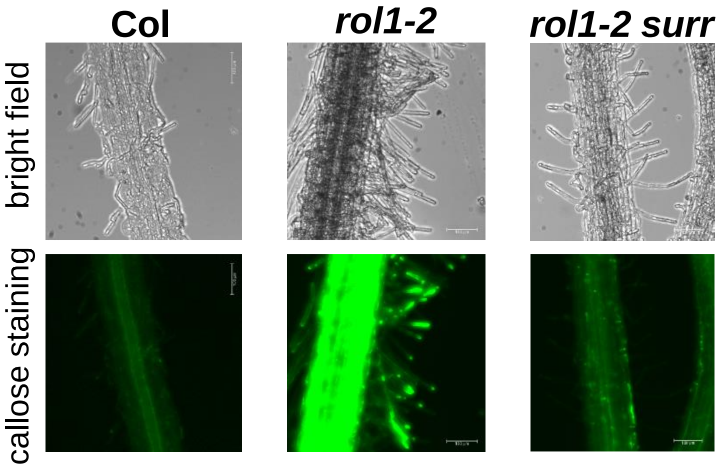

Suppl Figure S1 Ectopic deposition of callose in *rol1-2* roots.

Roots of seedlings were grown for 6 days and callose was stained with aniline blue. In contrast to the wild type, *rol1-2* roots revealed strong staining with aniline blue that was reduced in *rol1-2 surr* roots with only interspersed aniline staining. The overexposure was used to better visualize the remaining aniline staining in the *rol1-2 surr* double mutant. Bright field and fluorescence microscopic picture are shown on top and bottom row, respectively. Bar=100 µm.

Suppl Fig S2

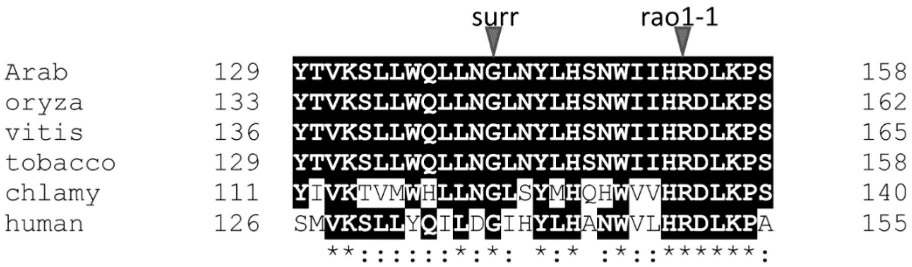

Suppl Figure S2 The *surr* mutation affects a conserved residue in CDK8.

CDK8 protein alignement of different species shows conservation of the Gly141 changed in the surr mutant to Glu. The species compared are *Arabidopsis thaliana* (Arab), *Oryza sativa* (Oryza), *Vitis vinivera* (Vitis), *Nicotiana tabacum* (tobacco), Chlamydomonas reinhardii (chlamy), *Homo sa*piens (human).
Asterisks indicate identical and colons highly similar residues in all species. Identical residues are also shown with black background.

Suppl Table S1

primers used for genotyping

Suppl Data Table S1

**Primer name**	**Primer sequence**	**allele, polymorphism**
rol1-2_F	TGAGGCGAACATCAAGTTTGTCGACTGCA	*rol1-2*
rol1-2_R	TGACTTGGTTTTTCAGATAAAGT	*Pst*I cuts wild type
cdk8_EMS_F2	GACTGCTATCCGCGAGATCATG	*surr*
cdk8_EMS_R2	TCCAATTACTGTGAAGATAGTTGAAT	*Eco*RI cuts mutant
flsl5_F	GATTGAGTACATCACTTCAGGTCCAATT	*ndpk2*
NDPK2_RT_R	CAATCTCACGCTTGCCGTTTTCAG	*Mfe*I cuts mutant
Man2_mut_F	GGTGAGTATATTTCCTGTCCAG	*man2*
Man2_mut_R	GAAGTGAATGAGCCAATAGCAG	*Nco*I cuts wild type
tetra_Mut_F2	CACCACATGTTCGACCATTTGTC	*tpr*
tetra_Mut_R	AAGAGAAGCTAGTGTCCTGTCCCAG	BstNI cuts wild type
rao1-1_F	TATCTTCACAGTAATTGGATTATCTGCA	*rao1-1*
rao1-1_R	TGCTTCGAACCAAGAAGCAGCTC	*Pst*I cuts wild type
rao1-2_F2	AAGACTCCGCCTAAAAGACCTAGT	*rao1-2*
rao1-2_R	ATAGAGATCGTACTCGGCATAATCAAA	*Rsa*I cuts mutant
ACTIN2F	AATGAGCTTCGTATTGCTCC	
ACTIN2R	GCACAGTGTGAGACACACC	

Primers used for genotyping. Underlined positions are mutated positions to introduce restriction site polymorphism between wild type and mutant.
